# Understanding veterinarians’ antibiotic choices and eliciting acceptable non-inferiority margins for randomized trials: a multi-country study

**DOI:** 10.1093/jacamr/dlag207

**Published:** 2026-09-21

**Authors:** J Buckell, T M Sørensen, J S Weese, L R Jessen, E M Broens, D Timofte, A F Bağcıgil, M C Nolff, D Singleton, D Verwilghen, A S Walker, F Allerton, K B Pouwels

**Affiliations:** Nuffield Department of Primary Care Health Sciences, University of Oxford, Oxford, UK; Department of Veterinary Clinical Sciences, University of Copenhagen, Frederiksberg, Denmark; Department of Pathobiology, Ontario Veterinary College, University of Guelph, Guelph, Canada; Department of Veterinary Clinical Sciences, University of Copenhagen, Frederiksberg, Denmark; Department of Biomolecular Health Sciences, Faculty of Veterinary Medicine, Utrecht University, Utrecht, The Netherlands; School of Veterinary Science, University of Liverpool, Neston, UK; Department of Veterinary Microbiology, Faculty of Veterinary Medicine, İstanbul University-Cerrahpaşa, Avcılar, İstanbul, Türkiye; Clinic for Small Animal Surgery, Vetsuisse Faculty, University of Zurich, Zurich, Switzerland; IVC Evidensia, Keynsham, Bristol, UK; Goulburn Valley Equine Hospital, Congupna, Victoria, Australia; Sydney School of Veterinary Science, The University of Sydney, Sydney, NSW, Australia; Nuffield Department of Medicine, University of Oxford, Oxford, UK; Willows Veterinary Centre and Referral Service Ltd, Shirley, Solihull, UK; Nuffield Department of Primary Care Health Sciences, University of Oxford, Oxford, UK

## Abstract

**Objectives:**

Unnecessary antibiotic use is a problem in both human and veterinary medicine. A clear understanding of prescribing behaviours and the underlying drivers is required to design studies that can provide the necessary evidence to identify areas or populations where antibiotics can safely be reduced.

**Methods:**

We conducted three discrete choice experiments to evaluate prescribing behaviour in different clinical scenarios where antibiotics are frequently part of case management: canine acute diarrhoea (CAD), post-operative prophylaxis (SAP), and urinary tract infection (UTI). We estimated acceptable non-inferiority margins for clinical trials (i.e. the maximum outcome loss clinicians would accept in exchange for reduced antibiotic use) for CAD and UTI. A total of 1219 small animal veterinarians across six global regions were surveyed.

**Results:**

Key drivers of prescribing behaviour included severity of clinical signs (CAD), anticipated clinical and microbiological cure rate (UTI), and risk of surgical site infection (SAP). Client pressure also impacted on prescribing decisions in all scenarios. In two of three scenarios, CAD and UTI, potentially acceptable non-inferiority margins were identified: 0–2 days for CAD duration without antibiotics; and 10% for clinical and microbiological cure in UTI cases. We found substantial geographical variation in prescribing habits, with higher rates in Asia and Southern/Eastern Europe.

**Conclusions:**

Results highlight problematic areas for targeting reduction measures but should support the development of antimicrobial stewardship guidelines aiming to reduce unnecessary antibiotic use.

## Introduction

Antimicrobial resistance (AMR) is a growing global health threat, with unnecessary antibiotic use in human and veterinary care making major contributions.^1^ While antibiotic use for livestock has been reduced substantially in many countries, inappropriate prescribing for companion animals is more persistent, especially for Highest Priority Critically Important Antimicrobials (HPCIAs) such as third-generation cephalosporins and fluoroquinolones.^2,3^

Reducing overuse of antibiotics among companion animals is not only important for animal health, it is also likely important for reducing AMR among humans. Unlike livestock, companion animals live in close contact with people, sharing homes and frequent affection, creating opportunities for bidirectional transmission of resistant pathogens, including MRSA and ESBL-producing *Escherichia coli*.^4^ Pet ownership is very common in high-income countries, with e.g. half of UK households owning a pet,^5^ and pet ownership is rising in several low- and middle-income countries.^6^ Combined with continued evidence of antibiotic overuse for conditions like acute diarrhoea,^4^ the potential contribution of this sector to global AMR is increasingly recognized, underscoring the need to improve antimicrobial stewardship for companion animals.

Antimicrobial stewardship guidelines aim to promote judicious antibiotic use in companion animal practice and are either evidence-based^7^ or from consensus statements.^8–10^ Randomized controlled trials (RCTs) represent the highest standard of clinical evidence.^11^ In the context of evaluating ways to reduce unnecessary use of existing antibiotics, most RCTs are designed as non-inferiority trials, reflecting the goal of safely reducing antibiotic use without impacting cure rates. In contrast to superiority trials, which aim to demonstrate that one intervention performs better than another, non-inferiority trials are designed to test whether a new or alternative strategy performs no worse than a standard approach by more than a prespecified, clinically acceptable (non-inferiority) margin. Non-inferiority margins are generally set by the trial investigators in a pragmatic manner, rather than by interrogating the guideline end user as to their actual preferences.^12^ Because the non-inferiority margin defines the outcome rate that is still acceptable, the trial’s conclusion hinges on where that is set and whether it is truly acceptable to veterinarians and animal owners. If the non-inferiority margin is too wide, a reduced-antibiotic strategy may be deemed non-inferior yet remain clinically unacceptable to practitioners, or potentially compromise patient care. Such results are less likely to engender change in prescribing. Therefore, non-inferiority margins should be established from practising veterinarians, the end users of stewardship guidance, to ensure clinical credibility and maximize adoption of trial findings.

This need is especially salient for canine acute diarrhoea (CAD), surgical antimicrobial prophylaxis (SAP), and presumptive urinary tract infections (UTIs; bacterial cystitis), common clinical scenarios where overuse or excessive duration of antibiotic courses are commonplace and where credible non-inferiority trials of stewardship interventions could change practice. Despite consistent guidance to the contrary,^13^ antimicrobial treatment is administered in 28%–70% of CAD cases.^14–17^ However, only a trivial reduction in the duration of CAD following antimicrobial treatment has been previously documented,^18^ and there is evidence that antimicrobials do not exert any beneficial effect in cases of CAD with mild or moderate illness.^19^ SAP is administered to prevent surgical site infections (SSIs)^20^ and accounts for a significant proportion of antimicrobial use in people^21^ and in pets.^22^ However, the morbidity-reducing benefits of SAP must be carefully balanced against the risks of adverse drug reactions and the promotional impact on AMR.^23^ The optimal duration of antimicrobial therapy to manage sporadic bacterial cystitis in female dogs has not been established and is currently based on expert opinion.^10^ A prospective study, designed to determine optimal course length, is underway^24^ using non-inferiority margins selected arbitrarily by the study authors, potentially misaligning with stakeholder preferences.

In this study, we aimed first, given the paucity of data on variation and drivers of antibiotic prescribing decisions in many countries, to evaluate the impact of drivers of choices, and variation between regions. Second, we aimed to establish acceptable non-inferiority margins for future trials of antimicrobial use in diarrhoea, sporadic cystitis and postsurgically.

Throughout the article, ‘presumptive UTI’ refers to the experimental scenario because at the point of treatment, the presence of bacterial cystitis is unknown. ‘Bacterial cystitis’ is used only when discussing evidence or guidance specific to that diagnosis and incorporates recently revised terminology.^25^

## Materials and methods

Ethical approval was granted from Royal College of Veterinary Surgeons (RCVS) Ethics Review Panel (ERP), Ref: 2021-81-Allerton.

### Sampling

To elicit preferences of small animal veterinarians, multiple strategies for recruitment were employed. These included recruitment through professional networks of advisory board members; through mailing lists of professional bodies such as the World Small Animal Veterinary Association (WSAVA) and the European Network for the Optimization of Veterinary Antimicrobial Therapy (ENOVAT); a promotional article in the monthly magazine of the British Small Animal Veterinary Association (BSAVA), *Companion*; and at three international veterinarian conferences.

We included small animal veterinarians who were currently practising in any country around the world. To increase accessibility, the survey was translated by veterinarians from English so that eight languages were available: English, Japanese, Mandarin, Portuguese, Romanian, Spanish, Turkish, and Vietnamese.

### Questionnaire measures and descriptive analysis

Before the Discrete Choice Experiments (DCEs), participants reported their country of current practice, country of veterinary training, years in practice after qualification, and gender. Whether country of practice differed from country of training was derived from the two country questions. After the DCEs, they reported the number of small animal veterinarians in their practice and answered questions about prescribing autonomy, client pressure and antimicrobial stewardship. Participant characteristics were summarized using counts and percentages for categorical variables, and medians and IQRs for continuous variables.

### Discrete Choice Experiment

A DCE is an experimental technique used widely in health sciences to understand preferences by asking participants to make experimentally controlled choices.^26^

Three DCEs assessed antibiotic prescribing in common scenarios: CAD, postoperative care following foreign body removal (SAP), and presumptive UTI. These are contexts where non-inferiority trials may be relevant. Scenarios were presented in increasing complexity to aid respondent familiarization. Design details for each experiment are provided in Tables 1–3.

**Table 1.** DCE: CAD: Choice for outpatient dog with diarrhoea

	No antibiotic	Antibiotic	Design edits
Likely duration of diarrhoea, after practice visit, based on your decision	0–1 (days more)^a^	4 days	
	0–2 (days more)		
	0–3 (days more)		
	0–4 (days more)		
Client attitude at practice visit	Follows your recommendation^a^		Restriction: must be the same for all options
	Insists on antibiotic		
Clinical signs at practice visit	Bright, alert, responsive (BAR), non-haemorrhagic diarrhoea^a^		Restriction: must be the same for all options
	Bright, alert, responsive (BAR), haemorrhagic diarrhoea		
	Mildly dehydrated, quiet but responsive, non-haemorrhagic diarrhoea		
	Mildly dehydrated, quiet but responsive, haemorrhagic diarrhoea		

^a^Denotes the reference level for each attribute (i.e. held constant in estimation).

**Table 2.** DCE: SAP: Choice around post-operative oral antibiotic for removal of a non-penetrating gastrointestinal foreign body

	No post-operative oral antibiotic	3 day post-operative oral antibiotic	7 day post-operative oral antibiotic	Design edits
Risk of surgical site infection^a^, based on your decision	0%–1% higher risk^b^	0%–1% higher risk^b^	5%	Restriction: 3 day antibiotic ≤ no antibiotic
	0%–2% higher risk	0%–2% higher risk		
	0%–3% higher risk	0%–3% higher risk		
	0%–4% higher risk	0%–4% higher risk		
	0%–5% higher risk	0%–5% higher risk		
Risk of surgical site infection with resistant bacteria, based on your decision	1 in 200	1 in 200^b^	1 in 200^b^	Restriction: antibiotic ≥ no antibiotic
		1 in 100	1 in 100	Restriction: 3 days ≤ 5 days
		1 in 50	1 in 50	
		1 in 33	1 in 33	
Client attitude at practice visit	Follows your recommendation^b^			Restriction: must be the same for all options
	Insists on antibiotic			
Probability of gastro/dermatological side effects, based on your decision	0%	1% 5%^b^	1% 5%^b^	Restriction: 3 days ≤ 7 days

^a^Higher risk than with a course of 7 days.

^b^Denotes the reference level for each attribute (i.e. held constant in estimation).

**Table 3.** DCE: UTI: Choice for outpatient dog with urinary tract infection (UTI)

	3 day course with a beta-lactam	5 day course with a beta-lactam	7 day course with a beta-lactam	Design edits
Likely clinical cure probability, based on your decision	65%–85%	65%–85%	85%	Restriction: 5 days ≥ 3days
	70%–85%	70%–85%		Restriction: 7 days ≥ 5 days
	75%–85%	75%–85%		
	80%–85%^a^	80%–85%^a^		
Likely microbiological cure probability, based on your decision	60%–80%	60%–80%	80%	Restriction: 5 days ≥ 3days
	65%–80%	65%–80%		Restriction: 7 days ≥ 5 days
	70%–80%	70%–80%		
	75%–80%^a^	75%–80%^a^		
Probability of gastrointestinal/dermatological side effects, based on your decision	1%^a^	1%^a^	5%	Restriction: 5 days ≥ 3days
	5%	5%		Restriction: 7 days ≥ 5 days
Client attitude at practice visit	Follows your recommendation^a^			Restriction: must be the same for all options
	Insists on longest duration			
Clinical signs at practice visit	None^a^			
	Moderate			Restriction: must be the same for all options
	Severe			

^a^Denotes the reference level for each attribute (i.e. held constant in estimation).

The first DCE (DCE: CAD; Table 1) described an outpatient dog with CAD, ‘A client presents at your practice with an adult dog with acute diarrhoea.’ Respondents chose between ‘No Antibiotic’ and ‘Antibiotic’. Attributes included clinical signs, client expectations, and expected diarrhoea duration conditional on treatment choice. Levels were informed by trial evidence and disease severity scales.^18,27,28^ Client expectation and clinical signs were held constant within each choice task but varied across tasks, reflecting real consultations where both the patient and client remain the same within a visit but differ between cases. An example task is shown in Figure S1 (available as Supplementary data at *JAC-AMR* Online).

The second DCE (DCE: SAP; Table 2) described a dog undergoing foreign body removal: ‘You have performed surgery to remove a non-penetrating gastrointestinal foreign body from an adult dog.’ Respondents chose between ‘No antibiotic’, ‘3-day’ or ‘7-day’ post-operative oral antibiotic courses. Attributes included risks of SSI and antibiotic-resistant SSI, client expectations, and gastrointestinal side effects, all conditional on prescribing decisions. Levels were based on surgical outcomes evidence.^29,30^ Client expectation was held constant within tasks, reflecting a single consultation. Constraints imposed on the design ensured that SSI risk decreased with longer treatment duration. An example is shown in Figure S2.

The third DCE (DCE: UTI; Table 3) described an outpatient female dog with a urinary tract infection: ‘You examine an adult female dog and suspect a urinary tract infection given the presence of the following clinical signs: pollakiuria, dysuria and haematuria. Pending urine culture results, you decide to prescribe a beta-lactam antibiotic (amoxicillin).’ Choices were between 3, 5 or 7 day beta-lactam courses. Attributes included clinical and microbiological cure, side effects, client expectations and clinical signs, with levels based on UTI evidence.^31^ As in other DCEs, client and patient characteristics were fixed within tasks but varied across scenarios (Figure S3).

Across DCEs, alternatives reflected clinical practice. DCE: CAD and DCE: SAP captured the decision to prescribe or not, while DCE: UTI examined treatment duration, consistent with guidelines recommending antibiotics for UTI. This design enabled assessment of both prescribing initiation and course length.

The survey comprised demographic questions about participants, followed by the three DCEs, and then questions about their practices. Attributes and levels were informed by published evidence and clinical input. The survey and experimental design were piloted with 20 veterinarians who were not included in the main sample. Pilot estimates were used as priors for the main Bayesian D-efficient designs. Following the pilot, the number of levels was reduced for the side effect attributes in the post-operative and UTI experiments, and for clinical signs in the UTI experiment. Participants were randomized to one of two experimental blocks of choice questions and completed six choice tasks per DCE, preceded by narrative instructions and a practice task. Technical experimental design details are presented in Supplementary Methods S1.

### Statistical analysis

Modelling sought to explain veterinarians’ experimental choices as a function of the attributes and demographic information collected in the survey. Choices were analysed using multinomial logit models. Utility for each alternative was specified as a linearly additive function of alternative-specific constants and dummy-coded attribute levels. Robust standard errors were computed using the sandwich estimator.

For each choice model (one for each DCE), coefficients were exponentiated and reported as ORs. Potential non-inferiority margins were identified using relevant estimated parameters. Predicted prescribing probabilities were then calculated for an illustrative veterinarian and clinical profile while varying the outcome attribute of interest. We examined more flexible random-parameter models and regional interactions for the attributes used to identify non-inferiority margins; these did not materially change either the central estimates or predictions, so the more parsimonious models are reported. Full utility specifications, sensitivity analyses and model estimates are provided in Supplementary Methods S2.

Veterinarian characteristics were incorporated as deterministic interactions with the prescribing alternative: gender, years in practice (continuous), whether the country of practice differed from the country of training, and geographical region. Years in practice was modelled continuously. In Figures 2–4, its OR shows the comparison of one veterinarian with five additional years of practice than another. Country of practice was grouped into 11 analytical categories: Africa, Asia, Australasia, Eastern Europe, North America, Northern Europe, South America, Southern Europe, the UK, Western Europe and “not reported”. Models used the UK as the reference category due to its large sample size and different legislative arrangements compared with the rest of Europe. Table 4 reports these categories, and Table S1 lists the allocation of countries to regions. To allow for possible differences in choice consistency between translated versions, the scale of utility was allowed to vary by survey language, with English as the reference. Using these models, it was possible to predict probabilities of prescribing antibiotics, evaluate variation in prescribing behaviours across demographics, and assess whether there were non-inferiority margins for the relevant outcomes—CAD duration, clinical cure and SSI risks—that were generally acceptable to practising small animal veterinarians. Technical details are provided in Supplementary Methods S2.

**Table 4.** Characteristics of the 1219 participating veterinarians

	*n*	%^a^	Median	IQR	Missing
*Characteristic*					
Gender					
Female	760	62.3			
Male	428	35.1			
Prefer not to answer	31	2.5			
Same country of practice and training	1091	89.5			
Different country of practice and training	128	10.5			
Years of practice			9	15	0
Number of vets in practice			4	6	189
*Geographical region*					
Africa	10	0.8			
Asia	332	27.2			
Australasia	48	3.9			
Eastern Europe	106	8.7			
North America	49	4.0			
Northern Europe	101	8.3			
South America	107	8.8			
Southern Europe	156	12.8			
UK	262	21.5			
Western Europe	47	3.9			
Not reported	1	0.1			
*Survey language*					
Chinese	288	23.6			
English	602	49.4			
Japanese	11	0.9			
Portuguese	25	2.1			
Romanian	81	6.6			
Spanish	106	8.7			
Turkish	103	8.4			
Vietnamese	3	0.2			
*DCE responses*					
Diarrhoea DCE	1219	100.0			
SAP DCE	1093	89.7			
UTI DCE	1042	85.5			

DCE, discrete choice experiment; SAP, surgical antimicrobial prophylaxis; UTI, urinary tract infection.

^a^Percentages use 1219 as the denominator unless missing values are shown.

## Results

### Sampling

**Figure 1.**
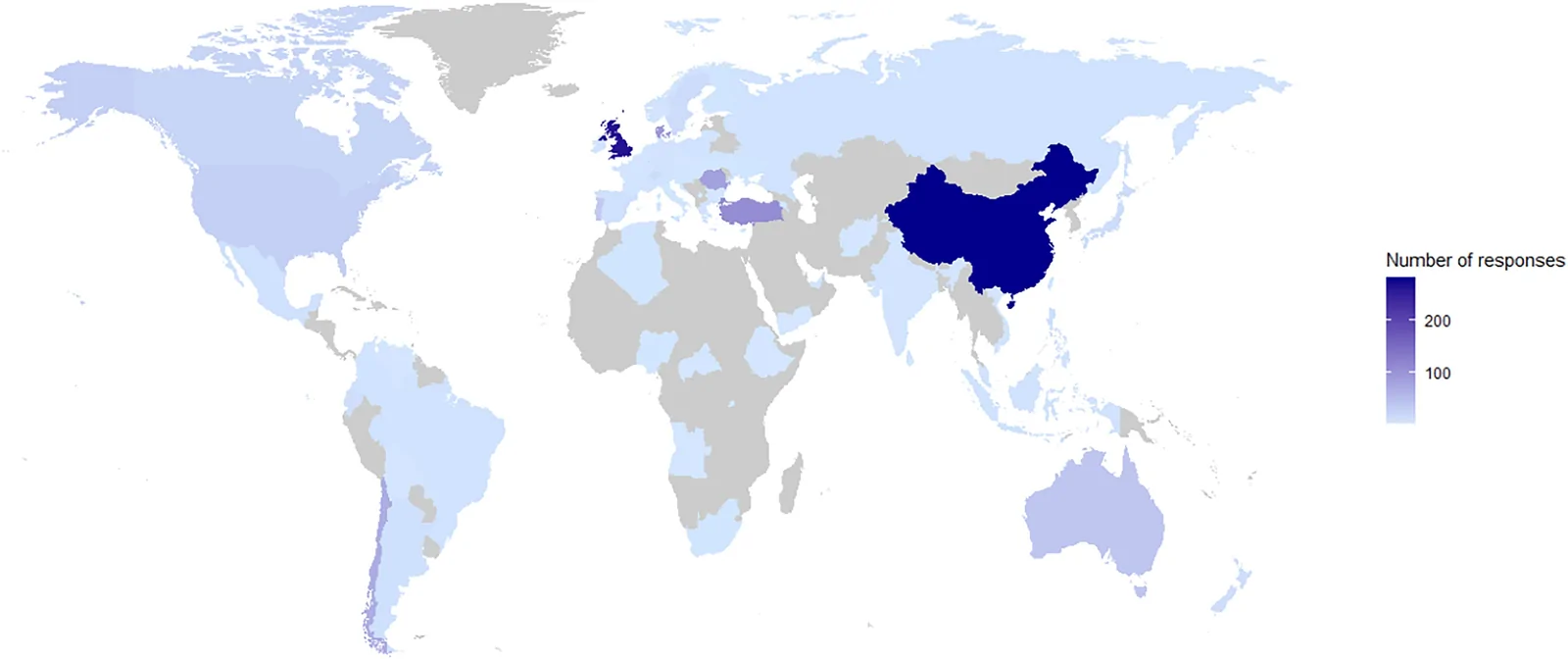
A total of 1219 vets from 71 countries. Darker shading denotes responses received from that country; grey shading denotes no response from that country.

In total, 2609 small animal veterinarians began the survey. Of these, almost half (*n* = 1390) did not progress as far as the first DCE. A small proportion (*n* = 198) completed at least one of the DCEs, but did not complete the full survey, whilst 1021 completed the full survey. Data from 1219 veterinarians (i.e. 1021 + 198) were modelled. Descriptive statistics are presented in Table 4. Figure 1 shows the geographical distribution of veterinarians sampled.

### Descriptive statistics

Respondents had a median of 9 years in practice (IQR 15) and worked in practices with a median of four veterinarians (IQR 6). Country of practice and training: same country, 1091 (89.5%); different countries, 128 (10.5%). The sample covered 71 countries; the largest analytical groups were Asia (*n* = 332) and the UK (*n* = 262). All 1219 respondents completed the CAD DCE, 1093 completed the post-operative DCE and 1042 completed the presumptive UTI DCE.

### DCE: CAD. Choice for outpatient dog with diarrhoea

**Figure 2.**
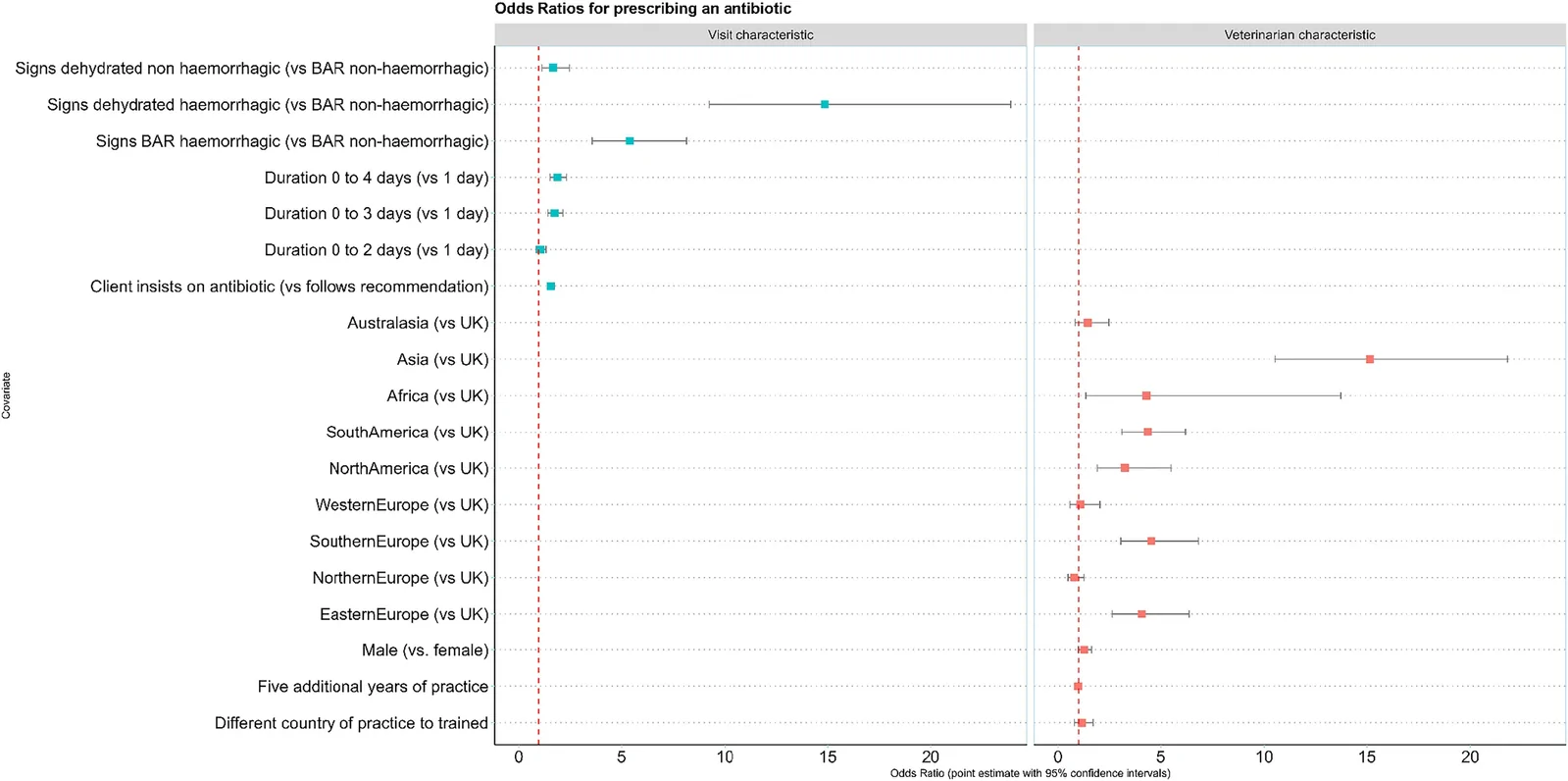
ORs for antibiotic prescriptions in the CAD experiment (i.e. the binary choice to prescribe an antibiotic or not to). The 95% CIs are computed analytically using the delta method. The left pane shows the experimentally varied features of visit characteristics. The right pane shows the veterinarian features (collected in the survey and used in modelling). Each covariate is presented separately on the *y*-axis; for categorically coded variables, the reference (i.e. omitted) category is shown in parentheses. BAR, bright, alert, responsive.

Full results from 1219 veterinarians are shown in Table S2, with ORs in Figure 2.

Antibiotic prescribing increased when diarrhoea duration exceeded the expected course under treatment (∼4 days). Prescribing was higher for 3 days versus 1 day (OR: 1.76; 95% CI: 1.44–2.16) and 4 days versus 1 day (OR: 1.90; 95% CI: 1.54–2.34), but not for 2 days versus 1 day (OR: 1.08; 95% CI: 0.87–1.34), suggesting a non-inferiority margin of 0–2 days.

All other attributes influenced prescribing. Clinical presentation had the largest effect: antibiotics were far more likely for dehydrated dogs with haemorrhagic diarrhoea than for bright, non-haemorrhagic cases (OR: 14.86; 95% CI: 9.24–23.88). Client insistence also increased prescribing (OR: 1.57; 95% CI: 1.41–1.75).

For CAD, prescribing varied by region: compared with the UK, odds of prescribing were higher in Africa (OR: 4.30; 95% CI: 1.35–13.74), Asia (OR: 15.15; 95% CI: 10.53–21.80), Eastern Europe (OR: 4.08; 95% CI: 2.62–6.37), Southern Europe (OR: 4.55; 95% CI: 3.03–6.82), North America (OR: 3.23; 95% CI: 1.90–5.50) and South America (OR: 4.37; 95% CI: 3.09–6.20). No differences were observed by gender, experience or whether the country of practice differed from the country of training.

### DCE: SAP. Choice for post-operative oral antibiotic (removal of a non-penetrating gastrointestinal foreign body)

**Figure 3.**
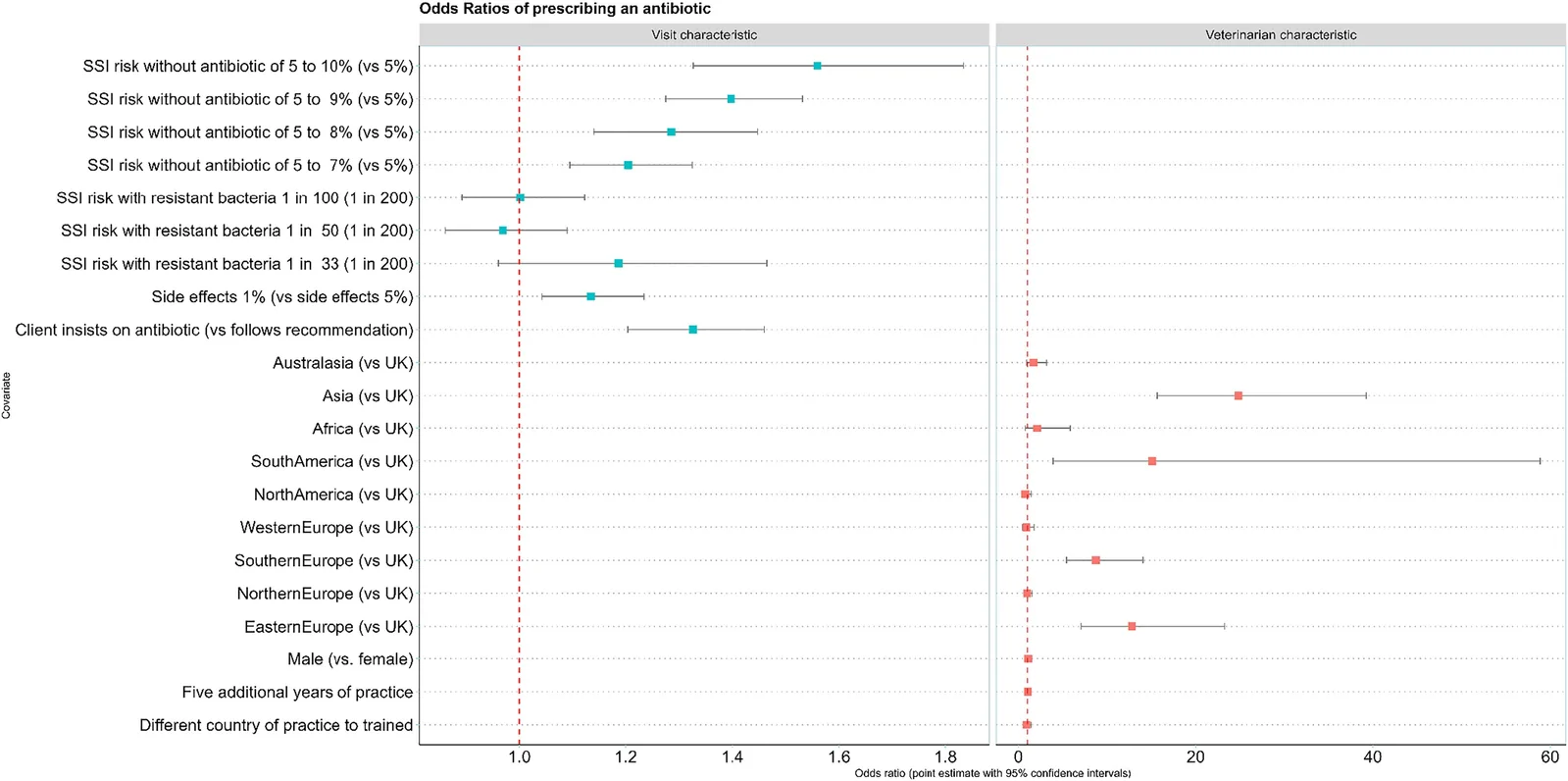
ORs for antibiotic prescriptions in the SAP experiment (i.e. the binary choice to prescribe an antibiotic or not to). The 95% CIs are computed analytically using the delta method. The left pane shows the experimentally varied features of visit characteristics. The right pane shows the veterinarian features (collected in the survey and used in modelling). Each covariate is presented separately on the *y*-axis; for categorically coded variables, the reference (i.e. omitted) category is shown in parentheses. SSI, surgical site infection.

Results from 1093 veterinarians are shown in Table S3. ORs for prescribing an antibiotic, according to attributes and by veterinarians’ characteristics, are presented in Figure 3. Note that the axes’ ranges in Figure 3 vary dramatically, because regional differences in prescribing propensity were substantially larger than the estimated effects of changes in attributes.

SSI risk with resistant bacteria did not influence decisions, but overall SSI risk did. Prescribing increased as estimated SSI risk without antibiotics rose, with the highest odds at 0%–5% versus 0%–1% (OR: 1.56; 95% CI: 1.33–1.83). As all levels differed from the reference, none were suitable as non-inferiority margins.

Client attitude and side effects also affected decisions. Prescribing increased when the client insisted on an antibiotic rather than following the veterinarian’s recommendation (OR: 1.33; 95% CI: 1.20–1.46), and when the risk of side effects was lower (1% versus 5%; OR: 1.13; 95% CI: 1.04–1.23).

For SAP, prescribing varied by region: compared with the UK, odds of prescribing were higher in Asia (OR: 24.79; 95% CI: 15.66–39.25), Eastern Europe (OR: 12.78; 95% CI: 7.03–23.23), Southern Europe (OR: 8.69; 95% CI: 5.38–14.06) and South America (OR: 15.10; 95% CI: 3.87–58.87). As before, no differences were observed by gender, experience or whether the country of practice differed from the country of training.

### DCE: UTI. Choice for outpatient dog with urinary tract infection

**Figure 4.**
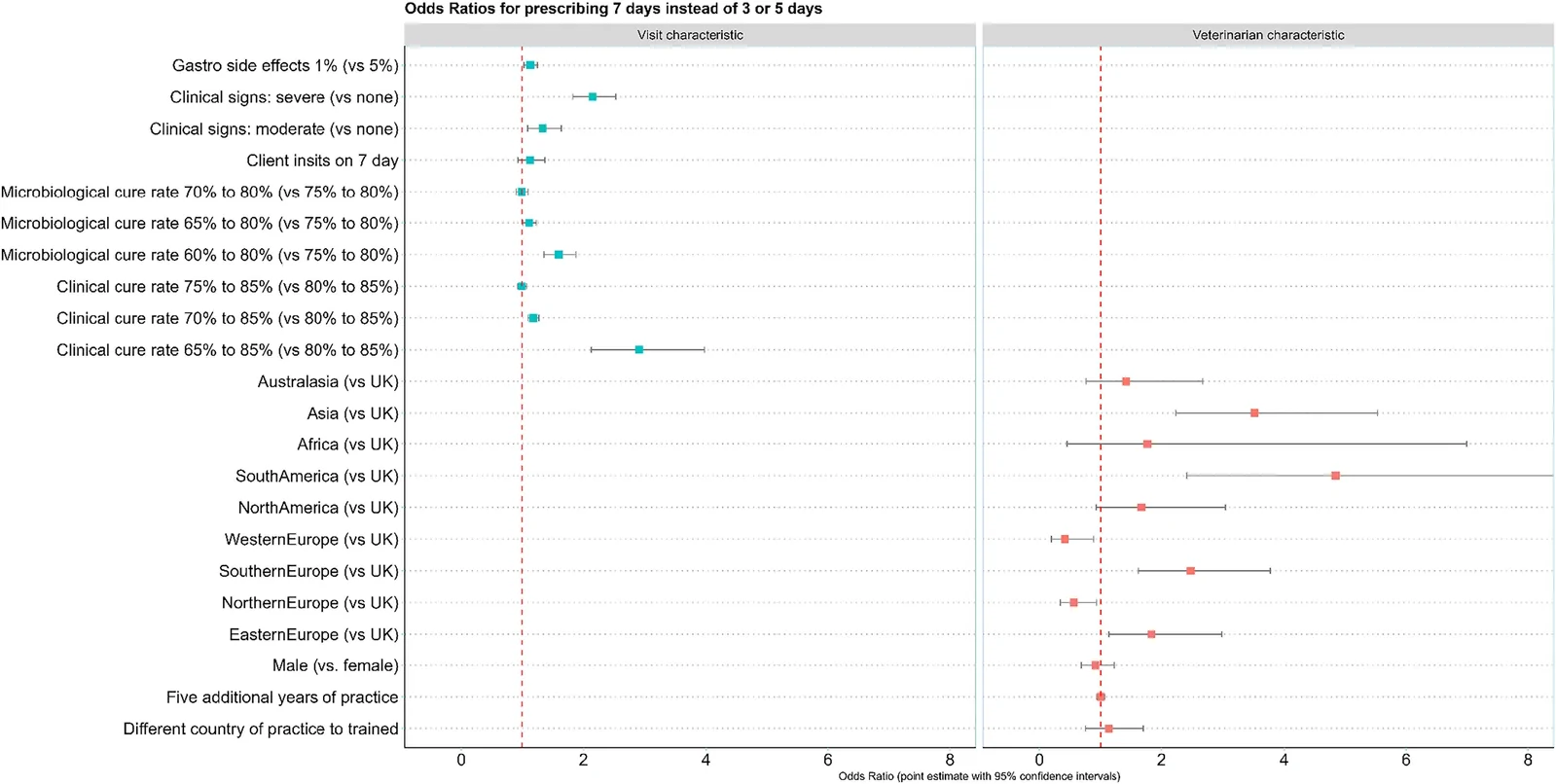
ORs for prescribing a 7 day course of antibiotics in the UTI experiment (i.e. 7 day versus 3 day or 5 day course). The 95% CIs are computed analytically using the delta method. The left pane shows the experimentally varied features of visit characteristics. The right pane shows the veterinarian features (collected in the survey and used in modelling). Each covariate is presented separately on the *y*-axis; for categorically coded variables, the reference (i.e. omitted) category is shown in parentheses.

Full results (*n* = 1042 veterinarians) are shown in Table S4; ORs for prescribing a 7 day course (versus 3–5 days) are in Figure 4.

Possible non-inferiority margins were derived from clinical and microbiological cure attributes, with the 7 day course fixed at 85% and 80%, respectively. The probability of prescribing 7 days increased as cure rates for shorter courses decreased, with statistically significant and monotonic effects for reductions >10% (but not 5%). A 10% difference therefore represents an appropriate non-inferiority margin for both outcomes.

Gastrointestinal side effects and clinical severity influenced prescribing. A lower risk of side effects with the longer course (1% versus 5%) increased the likelihood of a 7 day prescription (OR: 1.14; 95% CI: 1.03–1.25). Relative to no clinical signs, both moderate (OR: 1.34; 95% CI: 1.09–1.64) and severe clinical signs (OR: 2.15; 95% CI: 1.83–2.53) increased 7 day prescribing. Client attitude had no effect.

For UTI, prescribing varied by region: compared with the UK, odds of 7 day prescribing were higher in Asia (OR: 3.52; 95% CI: 2.23–5.53), Eastern Europe (OR: 1.84; 95% CI: 1.13–2.98), Southern Europe (OR: 2.47; 95% CI: 1.62–3.77) and South America (OR: 4.85; 95% CI: 2.41–9.75), and lower in Northern Europe (OR: 0.56; 95% CI: 0.34–0.93) and Western Europe (OR: 0.41; 95% CI: 0.19–0.88). No differences were observed by veterinarian characteristics.

### Predicting prescribing

**Figure 5.**
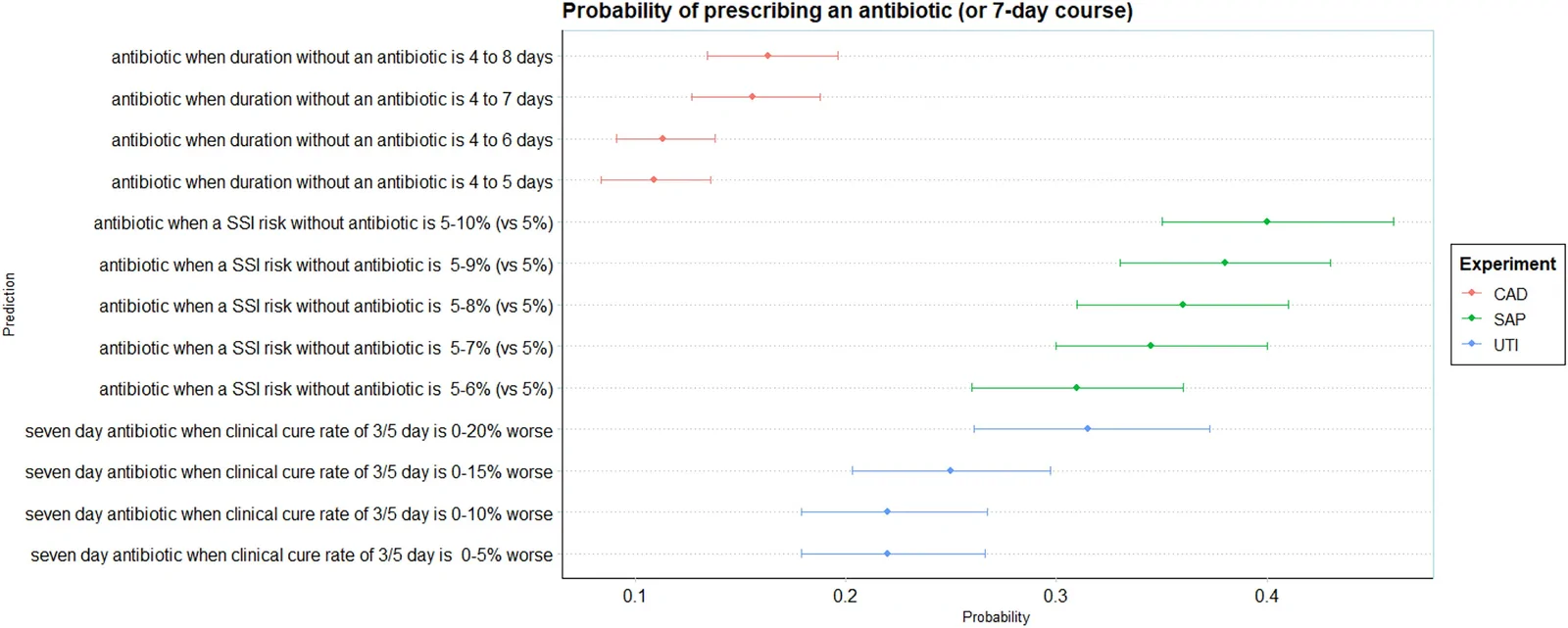
Predicted probabilities of antibiotic prescribing for the following experimental scenarios: CAD in an adult dog; post-operative prescribing after removal of a non-penetrating gastrointestinal foreign body from an adult dog (SAP); and course duration for a presumptive UTI in an adult female dog with compatible lower urinary tract signs pending urine-culture results.

The results of the predictions are shown in Figure 5.

For CAD, the probability of prescribing an antibiotic varied from 10.9% when the duration of disease was 4–5 days to 16.3% at 4–8 days. This suggests that longer perceived CAD duration in the absence of an antibiotic increases antibiotic use.

For SAP, antibiotic use probability increased as the estimated SSI risk without antibiotics rose above the 5% baseline. Mean prescribing probability increased from 31.0% at a 5%–6% SSI risk to 40.0% at a 5%–10% risk. This indicates a graded response of prescribing probability to absolute increases in perceived SSI risk.

For UTI, extending treatment to a 7 day antibiotic course became more likely as the clinical cure rate with shorter (3–5 day) therapy worsened. When the cure rate was 5% worse, the mean probability of a 7 day antibiotic course was 22.0%. This increased to 25.0% with a 15% decrement and then to 31.5% with a 20% decrement. This may have indicated a threshold effect, where modest differences in clinical cure did not strongly influence prescribing, but larger perceived losses in efficacy substantially increased the likelihood of longer antibiotic courses.

In all cases, uncertainty intervals overlapped substantially, meaning that predicted differences in prescribing probabilities were not different over the range of predictions.

## Discussion

This study recruited 1219 small animal veterinarians from a wide range of countries and elicited antibiotic prescribing behaviours for three common clinical scenarios encountered by small animal veterinarians. In two of three scenarios, CAD and UTI, potentially acceptable non-inferiority margins were identified, 0–2 days for CAD duration without antibiotics and 10% for clinical and microbiological cure in UTI cases. At these margins, we predicted that 11% of small animal veterinarians’ choices would be to prescribe an antibiotic for dogs with haemorrhagic CAD (these dogs would qualify for inclusion in a non-inferiority trial, the design of which we are aiming to inform). For UTI, we predicted that 22% of small animal veterinarians’ choices would be a 7 day antibiotic course over a 3 day or 5 day course for dogs with moderate clinical signs (these dogs would qualify for inclusion in a non-inferiority trial, the design of which we are aiming to inform). Higher rates of antibiotic use were observed in Asia, Eastern Europe and Southern Europe (compared with the UK). Variations across regions were evident for the three clinical scenarios; for the surgical setting, regional variation in prescribing far exceeded variation by clinical visit attributes. This was not the case for the remaining two scenarios, where variation was comparable across visit and veterinarian characteristics.

To our knowledge, this is the first study that shows that DCEs can be used to estimate which non-inferiority margins would be acceptable to veterinary clinicians. Although non-inferiority margins are traditionally justified from observed effects in previous randomized trials^32^ (if available) and clinical judgement, DCEs can complement estimates of the margins practising clinicians are likely to accept and hence act upon. Our findings that clinical severity strongly drives antibiotic prescribing for CAD, and that client pressure increases the chances of an antibiotic being given, are consistent with the literature.^33^ However, veterinarians also reported that it was increasingly rare in the UK for owners to ask directly for antibiotics, and if they did, most would accept the advice of the veterinarian if they were told the patient did not need antibiotics.^33–35^ The estimated acceptable non-inferiority margin was 10% for clinical/microbiological cure of UTI, which is also the non-inferiority margin used in the prospective trial of different antimicrobial treatment durations for presumptive canine UTIs.^24^ If a trial were to show that 3 days was non-inferior to 7 days with this margin, we predict that 78% of veterinarians would choose a 3 day duration, instead of the current default of 7 days, for a female dog with moderate clinical signs. Veterinarians were less likely to withhold post-operative antibiotics after removal of a non-penetrating gastrointestinal foreign body when the risk of overall SSI increased; interestingly, a higher risk of an antibiotic-resistant SSI with post-operative antibiotics did not influence the probability of providing post-operative antibiotics. This may be due to veterinarians perceiving antibiotic-resistant SSIs as being generally treatable with correctional surgery where the resistance profile is of less clinical significance or having a lack of experience with MDR SSIs.

The geographical prescribing pattern was not uniform across clinical scenarios and should not be interpreted as a single regional tendency to prescribe. The availability and content of antimicrobial stewardship guidelines vary between countries,^13^ as do patterns of antimicrobial purchasing within and between countries.^36^ Access to diagnostics, veterinary education and practice organization, and owner expectations may also influence prescribing decisions.^33^ Awareness and use of antimicrobial stewardship guidelines also vary across countries, and a European multinational survey found that countries with greater guideline awareness and country-specific guidance tended to report more appropriate antimicrobial use.^37^ These factors may contribute to regional differences in baseline prescribing, but were not measured directly here. Moreover, some regional estimates were dominated by one country—e.g. 281 of 332 respondents from Asia practised in China; and only 10 veterinarians were recruited in Africa, meaning results should not be considered as representative. Indeed, few reports on antimicrobial stewardship in companion animals are published from Asia or Africa. Studies here originate from high-income countries, e.g. Hong Kong^38^ and Singapore.^39^ This might reflect a lack of awareness and implementation of antimicrobial stewardship programmes in companion animals in low- and middle-income countries, as observed in human medicine, but reports for veterinary medicine are lacking to demonstrate this link.^40^ The regional results should therefore be treated as signals for further investigation and locally adapted stewardship, rather than estimates that apply uniformly to every country within a region.

Strengths of this study include using multiple DCEs, rather than just one. This meant that we were able to capture several common prescribing situations encountered by small animal veterinarians, giving a fuller account of their practice than if we had used a single DCE (as in all other veterinary cases). This further allowed us to estimate several non-inferiority margins of veterinarian antibiotic prescribing behaviour. This study used a large sample, comparable to large international surveys in veterinary medicine, and is the largest DCE conducted in veterinary medicine to date. A further strength is its geographical coverage, enabled in part by it being available in eight languages. Data were gathered from 71 countries across six World Organisation for Animal Health (WOAH) regions. The final models allowed the alternative-specific propensity to prescribe an antibiotic (or a 7 day course) to vary by region, while attribute coefficients were common across regions. In sensitivity analyses, we interacted region with the attributes used to identify the non-inferiority margins to test the robustness of our findings across region. No clear evidence of regional variation in these effects was found. We therefore retained the common attribute coefficients.

Limitations include that the DCE was a stated preference study and potentially vulnerable to hypothetical bias.^41^ We took steps to mitigate this bias, such as using the cheap talk approach^42^ and by using, with consent, the logos of two major veterinary bodies (WSAVA and ENOVAT) on the landing page of the survey. This endorsement gave the survey legitimacy and in turn encouraged truthful responses.^43^ Further evidence suggests that stated preference studies are less prone to hypothetical bias when the choices are well known to respondents and well described by their attributes.^44^ In health settings, a high concordance between stated preference responses and subsequent real-world decisions has been documented.^45^ Other major advantages of the stated preference approach include that we were able to consistently measure the outcomes across all regions without measurement error (e.g. observational data on prescribing could be measured on different scales in different countries and/or could be prone to misreporting), and the DCE was able to estimate the causal impact of the attributes on (experimental) choices; this is not necessarily the case in real-world settings where measured associations may be biased (e.g. by confounding). However, the extent to which any such bias can be eliminated is not clear. The scenarios necessarily simplified clinical presentations. In the CAD scenario, vaccination status and some physical examination findings were omitted. Veterinarians may therefore have made different assumptions about these unobserved features, reflecting both local practice patterns and individual clinical experience, and the estimates should be interpreted as applying to the information presented in the choice tasks. The post-operative scenario described a non-penetrating gastrointestinal foreign body but did not provide further operative detail or define the procedure. The results may not be generalized to penetrating foreign bodies, peri-operative contamination, bowel compromise or other features that alter SSI risk. The UTI scenario did not specify whether this was a first or recurrent episode, whether point-of-care urinalysis or sediment examination had been performed, or whether bacterial cystitis was subsequently confirmed. The findings therefore concern prescribing under the stated diagnostic uncertainty, which is likely reflective of the management approach in primary care practice. Nonetheless, it should not be interpreted as assuming that all cystitis is bacterial, that recurrent UTIs are managed identically to first occurrence, or that a positive culture alone establishes a UTI. Whilst we had good geographical variation, we were less able to recruit from Africa, where we solicited responses from 10 small animal veterinarians only. Relatedly, whilst we had a good spread of responses across regions, we had limited variation within regions. For example, 281 of the 332 respondents in Asia were from China; the next highest number of respondents in Asia was Japan (*n* = 11). We do not know the number of veterinarians contacted and, hence, the response rate to the survey.

These results can be used to inform non-inferiority margins for future randomized clinical trials for prescribing antibiotics in canine veterinary medicine. Moreover, the results can be used to inform the design of interventions targeting modifiable drivers of antibiotic use. For example, seeing the impact of client pressure, interventions targeting client knowledge or communication skills of veterinarians could be tested in future trials. The approach of using DCEs for estimating potentially acceptable non-inferiority margins can be adopted in other settings across both veterinary and human medicine. The results also highlight substantial scope for antibiotic stewardship interventions in regions where veterinarians were much more likely to choose to prescribe antibiotics and/or long courses for these clinical scenarios, particularly in Asia, Southern Europe and Eastern Europe.

In conclusion, experimental measurement of prescribing behaviours can inform non-inferiority margins for randomized non-inferiority trials and intervention design and targeting in small animal veterinary practice.

## Supplementary Material

dlag207_Supplementary_Data

## Supplementary data

Supplementary Methods S1 and S2, Figures S1 to S3, and Tables S1 to S4 are available as Supplementary data at *JAC-AMR* Online.
