# Supplementary material for: Understanding veterinarians’ antibiotic choices and eliciting acceptable non-inferiority margins for randomized trials: a multi-country study
Source: JAC Antimicrob Resist. 2026 Sep 21;8(5):dlag207. doi: 10.1093/jacamr/dlag207 (PMC13590232; doi:10.1093/jacamr/dlag207)
Supplement: Supplementary file 1 [file dlag207_supplementary_data.docx]

Supplementary data

Supplementary Methods S1: Experimental design

Pilot study and experimental design

A Bayesian D-efficient design was used to generate sets of choice tasks for all three experiments (Hensher et al., 2015). We first conducted a pilot study with 20 veterinarians, who were not included in the final sample. A multinomial logit (MNL) model was estimated using the pilot data to obtain parameter priors for the main design and to inform the sample-size calculation (de Bekker-Grob et al., 2015). Following the pilot, the probability of side effects was reduced from three levels to two in DCE SAP and DCE UTI, and clinical signs were reduced from four levels to three in DCE UTI. The updated experimental design (i.e., the choice-task combinations) were then regenerated using the pilot-informed priors and the revised attribute-level structure. Thus, the MNL model used at this stage informed design construction and sample-size planning, rather than serving as the final analysis model.

Main study design

The final design consisted of 12 choice tasks per DCE, arranged in two blocks of six tasks. This two-block structure was used for each of the three DCEs. Each respondent completed six choice tasks in each experiment, balancing concerns about learning and respondent fatigue (Hess et al., 2012). To reduce fatigue and support respondents' understanding, the order of the DCEs was from the simplest to the most complicated: CAD, SAP, and UTI. Before the experiment, respondents were given narrative information describing the choice tasks, attributes, and levels. A practice choice scenario provided step-by-step instructions for completing the tasks. The final MNL models were estimated using the main-study responses. The sample size exceeded the minimum required for statistical power based on the pilot parameter estimates.

Figure S1: DCE: CAD example choice task

Figure S2: DCE: SAP example choice task

Figure S3: DCE: UTI example choice task

Supplementary Methods S2: Statistical analysis

*Utility function*

Experimental choices were analysed using Multinomial Logistic (Logit) regression models. Based on McFadden (1974), respondents were assumed to maximise their utility in making choices. In this formulation, the veterinarian reconciles their preferences for each of the available alternatives and chooses that which maximises their utility; that is, choosing their preferred antibiotic prescription decision in the choice tasks. Veterinarians’ utility is assumed to be a linearly-additive function,

$U_{nti}= V_{nti}+\varepsilon_{nti}=\sum_{m} \beta_{m}\cdot x_{ntim}+\varepsilon_{nti}$	(1)

where: $U_{nti}$is the utility for veterinarian *n* for option *i* in task *t*, comprising deterministic and random utility; $V_{nti}$ is the deterministic component of utility; $\varepsilon_{nti}$ is the random component of utility; $x_{ntim}$ is the *m^th^* attribute-level of option *i*; and $\beta_{m}$ is the *m^th^* preference parameter to be estimated. The deterministic component of utility comprises alternative-specific constants (ASC) and attribute levels (dummy-coded).

Taking the Diarrhoea DCE as an example, we have:

$V_{d, nti}+\varepsilon_{d, nti}= {ASC}_{antibiotic}\cdot antibiotic+ \Sigma_{L-1=3}\beta_{duration}\cdot{duration}_{d,nti}+ \Sigma_{L-1=1}\beta_{client attitude}\cdot{client attitude}_{d,nti}+ \Sigma_{L-1=3}\beta_{clinical signs}\cdot{clinical signs}_{d,nti}+\varepsilon_{d, nti}$	(2)

Where $antibiotic$ is a binary variable that takes the value of 1 for the “antibiotic” alternative in the choice task and 0 for the “no antibiotic” alternative; ${ASC}_{antibiotic}$ is an “alternative-specific constant” (ASC) for antibiotics, and measures the preference for prescribing an antibiotic (versus not prescribing the antibiotic). *L* is the total number of levels for a given attribute. Since the attributes are dummy coded, one is held constant, and there are *L-1* parameters estimated. To illustrate, for the attribute $clinical signs$, there are four levels, *L=4*. Holding the reference category to zero, we then estimate parameters for the remaining, *L-1 = 3*, levels. Setting “Bright alert responsive (BAR), non-haemorrhagic diarrhoea” as the reference category, we estimate parameters for 3 levels, “Bright alert responsive (BAR), haemorrhagic diarrhoea”, “Mildly dehydrated, quiet but responsive, non-haemorrhagic diarrhoea”, and “Mildly dehydrated, quiet but responsive, haemorrhagic diarrhoea”. These parameters measure the preferences for each of those levels relative to the reference category. More specifically, the parameter on the level “Mildly dehydrated, quiet but responsive, haemorrhagic diarrhoea” measures the extent to which veterinarians are more likely to prescribe an antibiotic if the patient presents with those symptoms versus if the patient presents with the symptoms of “Bright alert responsive (BAR), non-haemorrhagic diarrhoea”. In Table 1, the reference level for each attribute in both DCEs is shown with *.

Assuming a type-I extreme value error distribution on the error term results in a multinomial logit (MNL) model for estimating choice probabilities,

$P_{d, nti}=\frac{exp(V_{d,nti})}{\sum_{j=1}^{J} exp(V_{d,ntj})}$,	(3)

Where J is the total number of alternatives.

*Preference heterogeneity*

Heterogeneous antibiotic prescribing preferences were modelled deterministically using veterinarians’ characteristics (gender, years of practice, whether the country of practice is the country of training, and geographical region).

Incorporating deterministic heterogeneity, taking ${ASC}_{antibiotic}$ in (1) above as an example, we have,

${ASC}_{antibiotic}={ASC}_{antibiotic,base}+{ASC}_{antibiotic,female}\cdot\left( {female}_{n} \right)+{ASC}_{antibiotic,experience}\cdot\left( {years of practice}_{n} \right)+{ASC}_{antibiotic,practice\_trained}\cdot\left( {country of practice and training}_{n} \right)+\Sigma_{R-1=10}{ASC}_{antibiotic,region}\cdot\left( {region}_{n} \right)$	(4)

Where ${ASC}_{antibiotic,base}$ is the base ASC for prescribing an antibiotic. ${female}_{n}$ is a binary variable that takes the value of 1 if the veterinarian is female and 0 otherwise; ${ASC}_{antibiotic,female}$ measures differences in prescribing probabilities between female and non-female veterinarians. ${years of practice}_{n}$ is a continuous variable of the reported number of years of practice for each veterinarian; ${ASC}_{antibiotic,experience}$ measures if prescribing probability differs as years of practice increases. ${country of practice and training}_{n}$ is a binary variable that takes the value of 1 if the veterinarian practices in the country they trained in and 0 otherwise; ${ASC}_{antibiotic,practice\_trained}$ measures whether prescribing probability differs if veterinarians practice in a different country to which they trained (versus those that practice in the country in which they trained). ${region}_{n}$ is a categorical variable for the veterinarian’s region; R is the total number of regions (R=11); $\Sigma_{R-1=10}{ASC}_{antibiotic,region}$ are 10 region parameters each measuring differences in prescribing probabilities between each region and the UK (the UK being the reference region).

Model parameters are exponentiated to yield odds ratios with corresponding standard errors.

*Scale heterogeneity*

Given that the experiment was administered in 8 languages, we wish to account for this in analyses. More specifically, the way that the experiment is described and is interpreted may differ subtly between languages. As a result, choice making could be slightly more or less deterministic due to these differences. We are able to model this explicitly through the scale of utility (which governs how deterministic choices are; Buckell and Hess, 2019); allowing the scale to vary across languages captures such effects.

$P_{d, nti}=\frac{exp({\sigma V}_{d,nti})}{\sum_{j=1}^{J} exp(\sigma V_{d,ntj})}$,	(5)

Where $\sigma$ is the scale of utility which multiplies the deterministic component of utility, $V_{d, nti}$. In turn,

$$\sigma=\Sigma_{La-1=7}\sigma_{language}\cdot\left( {language}_{n} \right)$$

${language}_{n}$ is a categorical variable for the language of the survey for veterinarian, *n*; *La* is the total number of languages (La=8); $\Sigma_{La-1=7}\sigma_{language}$ are 7 language scale parameters each measuring scale variation between each language and English (English being the reference language).

*Testing and sensitivity analyses*

We estimated models with random parameters that allow for unobserved preference heterogeneity. The estimated means of the distributions were very similar to those from the simpler models, and predicted probabilities were also very similar. However, the additional variation in the random-parameters models meant that preference distributions overlapped across levels. For the duration attribute in CAD, for example, this implies that some veterinarians' preferences could have been non-monotonic (that is, they preferred a worse duration to a better one). For these reasons, and to keep the results easier to interpret, we used the simpler model described above. Fit statistics for the three final models are reported with the model estimates below.

We tested for preference variation by geographical region for the non-inferiority margin attributes. This involved interacting the levels of attributes with dummy variables for regions. We did not find evidence to suggest variation in preferences for attributes related to non-inferiority margins and therefore these interactions were not included in the final models.

*Predicting prescribing of antibiotics*

We used the fitted model to compute the probability of veterinarians prescribing an antibiotic (or in the case of UTI, the probability of prescribing the longest course of 7 days, versus 3-day or 5-day).

We did so assuming a model veterinarian, i.e., the by using most common characteristics for veterinarians (female, 10 years of practice, trained in their country of practice, based in the UK).

We next assumed a baseline typical scenario for each condition, based on the clinical opinion. For diarrhoea, we assumed no client pressure, and the patient is bright, alert, and responsive, though with haemorrhagic diarrhoea. For UTI, we assumed a minimal microbiological cure probability of 75-80%, minimal gastrointestinal side effects (1%), no perceived client pressure, and moderate clinical signs at the practice visit. For SSI, we assumed the overall SSI risk to be minimal (5%), no perceived client pressure, and minimal gastrointestinal side effects (0%).

We then varied the remaining attribute in each case to examine the response in prescribing probabilities to changes in that attribute. For diarrhoea, the duration of diarrhoea without an antibiotic was varied from 0 to 1 days more to 0-4 days more. For SSI, the risk of a SSI without an antibiotic was varied from 0-1% higher risk to 0-5% higher risk. For UTI, clinical cure probability of a shorter course (either 3 or 5 days versus 7 days) being lower was varied from 80-85% to 65-85%.

*Code and data availability*

Data were analysed using the *Apollo* package in R (Hess and Palma, 2019). Data and analytical code are available on reasonable request.

| Geographical region | Countries assigned to region |
| --- | --- |
| Africa | Algeria, Angola, Central African Republic, Ethiopia, Nigeria, Rwanda, South Africa |
| Asia | Afghanistan, Burma, China, Hong Kong, India, Indonesia, Japan, Macao, Malaysia, Mauritius, Philippines, Singapore, Sri Lanka, Taiwan, United Arab Emirates, Vietnam, Yemen |
| Australasia | Australia, New Zealand |
| Eastern Europe | Armenia, Bulgaria, Croatia, Czech Republic, Hungary, Kosovo, Lithuania, Poland, Romania, Russia, Slovakia, Slovenia, Ukraine |
| North America | Canada, United States |
| Northern Europe | Denmark, Faeroe Islands, Finland, Norway, Sweden |
| South America | Argentina, Bolivia, Brazil, Chile, Colombia, Ecuador, Grenada, Mexico, Venezuela |
| Southern Europe | Cyprus, Greece, Italy, Portugal, Spain, Turkiye |
| UK | United Kingdom, Isle of Man |
| Western Europe | Austria, Belgium, France, Germany, Ireland, Netherlands, Switzerland |
| Not reported | Not reported |

Table S1: allocation of countries to regions.

References

Buckell J, Hess S. Stubbing out hypothetical bias: improving tobacco market predictions by combining stated and revealed preference data. Journal of Health Economics. 2019;65:93-102.

Bunch DS, Gay DM, Welsch RE. Algorithm 717: Subroutines for maximum likelihood and quasi-likelihood estimation of parameters in nonlinear regression models. ACM Trans Math Softw. 1993;19(1):109–30.

de Bekker-Grob EW, Donkers B, Jonker MF, Stolk EA. Sample Size Requirements for Discrete-Choice Experiments in Healthcare: a Practical Guide. The Patient - Patient-Centered Outcomes Research. 2015;8(5):373-84.

Hensher DA, Rose JM, Greene W. Applied Choice Analysis. Cambridge: Cambridge University Press; 2015.

Hess S, Hensher DA, Daly A. Not bored yet – Revisiting respondent fatigue in stated choice experiments. Transportation Research Part A: Policy and Practice. 2012;46(3):626-44.

Hess S, Palma D. Apollo: A flexible, powerful and customisable freeware package for choice model estimation and application. Journal of Choice Modelling. 2019;32:100170.

Langlois, D. K., et al. (2020). "Metronidazole treatment of acute diarrhea in dogs: A randomized double blinded placebo-controlled clinical trial." Journal of Veterinary Internal Medicine 34(1): 98-104.

McFadden,D.,1974.Conditional logit analysis of qualitative choice behavior. In: Frontiers in Econometrics, pp.105 142.

Train, K. 2009. Discrete Choice Methods with Simulation. Cambridge University Press, University of California, Berkeley

Supplementary results: Model estimates

|  | Estimate | Std.err. | t-ratio(0) | Rob.std.err. | Rob.t-ratio(0) |
| --- | --- | --- | --- | --- | --- |
| scale_english | 1 | NA | NA | NA | NA |
| scale_spanish | 1.248122901 | 0.126604949 | 9.858405278 | 0.158519344 | 7.873631514 |
| scale_portuguese | 1.703204508 | 0.295932706 | 5.755377743 | 0.37956447 | 4.487260114 |
| scale_chinese | 0.55979463 | 0.056156461 | 9.968481325 | 0.082053802 | 6.822287486 |
| scale_japanese | 1 | NA | NA | NA | NA |
| scale_turkish | 0.535642881 | 0.095237637 | 5.624277296 | 0.147733152 | 3.625745982 |
| scale_vietnamese | 1 | NA | NA | NA | NA |
| scale_romanian | 0.942512129 | 0.114145989 | 8.257076194 | 0.146387822 | 6.438459936 |
| antibiotic | -4.20648541 | 0.24636296 | -17.0743419 | 0.278448303 | -15.1068811 |
| antibiotic_male | 0.235416777 | 0.075358209 | 3.123969898 | 0.126871486 | 1.855553084 |
| antibiotic_years_practice | -0.00736717 | 0.003399157 | -2.16735355 | 0.006061362 | -1.21543218 |
| antibiotic_diff_country_practice_to_trained | 0.144457173 | 0.112074455 | 1.288939324 | 0.195350314 | 0.739477557 |
| antibiotic_Africa | 1.459757835 | 0.330427055 | 4.417791503 | 0.592242889 | 2.464795884 |
| antibiotic_Asia | 2.718072719 | 0.121304203 | 22.40707793 | 0.185737454 | 14.6339506 |
| antibiotic_Australasia | 0.35587574 | 0.18564144 | 1.917005924 | 0.278339465 | 1.278567306 |
| antibiotic_NorthernEurope | -0.25350285 | 0.15869823 | -1.59738929 | 0.246927544 | -1.02662849 |
| antibiotic_EasternEurope | 1.40701991 | 0.130386331 | 10.79116115 | 0.226581645 | 6.209770027 |
| antibiotic_SouthernEurope | 1.514956287 | 0.13071398 | 11.58985659 | 0.206881923 | 7.32280647 |
| antibiotic_WesternEurope | 0.079580071 | 0.202252051 | 0.393469785 | 0.321547514 | 0.247490861 |
| antibiotic_NorthAmerica | 1.172231636 | 0.165421843 | 7.086317117 | 0.271412008 | 4.319011697 |
| antibiotic_SouthAmerica | 1.475299085 | 0.115355442 | 12.78915891 | 0.177894379 | 8.293118035 |
| antibiotic_Other | 0 | NA | NA | NA | NA |
| no_antibiotic | 0 | NA | NA | NA | NA |
| duration_0_to_1 | 0 | NA | NA | NA | NA |
| duration_0_to_2 | -0.07774084 | 0.109300311 | -0.71125913 | 0.110910346 | -0.70093411 |
| duration_0_to_3 | -0.5676109 | 0.109900273 | -5.16478153 | 0.104324744 | -5.44080798 |
| duration_0_to_4 | -0.64092285 | 0.110396055 | -5.80566807 | 0.105850438 | -6.05498533 |
| client_follows_recommendation | 0 | NA | NA | NA | NA |
| client_insists_antibiotic | -0.45198324 | 0.074453264 | -6.07069741 | 0.054824684 | -8.24415578 |
| signs_BAR_non_haemorrhagic | 0 | NA | NA | NA | NA |
| signs_BAR_haemorrhagic | -1.68666671 | 0.199966804 | -8.43473352 | 0.209210078 | -8.06207199 |
| signs_dehydrated_non_haemorrhagic | -0.52631263 | 0.192109591 | -2.73964788 | 0.195417527 | -2.6932724 |
| signs_dehydrated_haemorrhagic | -2.69853809 | 0.215767284 | -12.5067065 | 0.242170505 | -11.1431328 |

Table S2: Estimates from DCE: CAD. Std.err - standard error, t-ratio(0) – t-ratio versus 0, Rob.std.err - robust standard error, Rob t-ratio(0) – robust t-ratio versus 0. Number of respondents = 1,219; final log-likelihood = -3,635.46; null log-likelihood = -5,069.68; AIC = 7,320.92; BIC = 7,493.36; McFadden's pseudo-R-squared = 0.2829.

|  | Estimate | Std.err. | t-ratio(0) | Rob.std.err. | Rob.t-ratio(0) |
| --- | --- | --- | --- | --- | --- |
| scale_english | 1 | NA | NA | NA | NA |
| scale_spanish | 0.45171467 | 0.11938314 | 3.78373912 | 0.24593758 | 1.83670454 |
| scale_portuguese | 1 | NA | NA | NA | NA |
| scale_chinese | 1 | NA | NA | NA | NA |
| scale_japanese | 1 | NA | NA | NA | NA |
| scale_turkish | 1 | NA | NA | NA | NA |
| scale_vietnamese | 1 | NA | NA | NA | NA |
| scale_romanian | 1 | NA | NA | NA | NA |
| no_antibiotic | 0 | NA | NA | NA | NA |
| three_day_antibiotic | -1.7418804 | 0.11310174 | -15.401005 | 0.1793689 | -9.7111613 |
| seven_day_antibiotic | -1.945465 | 0.11805059 | -16.479926 | 0.17811555 | -10.922488 |
| antibiotic_male | 0.07288801 | 0.07857407 | 0.92763436 | 0.16429104 | 0.44365176 |
| antibiotic_years_practice | 0.01062337 | 0.00317958 | 3.34112461 | 0.00716848 | 1.48195543 |
| antibiotic_diff_country_practice_to_trained | -0.110322 | 0.09955926 | -1.108104 | 0.23129692 | -0.4769714 |
| antibiotic_Africa | 0.73529633 | 0.26938322 | 2.72955511 | 0.52359825 | 1.40431396 |
| antibiotic_Asia | 3.21040139 | 0.11404638 | 28.1499639 | 0.23441315 | 13.6954836 |
| antibiotic_Australasia | 0.5265588 | 0.14384982 | 3.6604758 | 0.31860573 | 1.65269719 |
| antibiotic_NorthernEurope | -0.0456978 | 0.1089277 | -0.4195239 | 0.2353216 | -0.1941928 |
| antibiotic_EasternEurope | 2.54800863 | 0.14927451 | 17.0692814 | 0.30490991 | 8.35659498 |
| antibiotic_SouthernEurope | 2.16265726 | 0.11585324 | 18.6672149 | 0.24526426 | 8.8176617 |
| antibiotic_WesternEurope | -0.1124784 | 0.15394901 | -0.7306211 | 0.34171194 | -0.3291614 |
| antibiotic_NorthAmerica | -0.2774177 | 0.14355125 | -1.9325342 | 0.32179059 | -0.8621063 |
| antibiotic_SouthAmerica | 2.71469779 | 0.35325177 | 7.68488088 | 0.69416223 | 3.91075409 |
| antibiotic_Other | 0 | NA | NA | NA | NA |
| SSI_risk_0_to_1 | 0 | NA | NA | NA | NA |
| SSI_risk_0_to_2 | -0.1860336 | 0.06769149 | -2.7482566 | 0.04837282 | -3.8458287 |
| SSI_risk_0_to_3 | -0.2508222 | 0.08700216 | -2.8829427 | 0.06083259 | -4.1231556 |
| SSI_risk_0_to_4 | -0.334465 | 0.07360511 | -4.5440465 | 0.04691766 | -7.1287667 |
| SSI_risk_0_to_5 | -0.4442336 | 0.11226968 | -3.9568435 | 0.08266004 | -5.374224 |
| SSI_risk_bac_res_1_200 | 0 | NA | NA | NA | NA |
| SSI_risk_bac_res_1_100 | 0.00143983 | 0.07164553 | 0.02009659 | 0.05867852 | 0.02453761 |
| SSI_risk_bac_res_1_50 | -0.0311838 | 0.08007377 | -0.3894381 | 0.06005837 | -0.5192244 |
| SSI_risk_bac_res_1_33 | 0.17099561 | 0.13030658 | 1.31225611 | 0.10748386 | 1.59089569 |
| client_follows_recommendation | 0 | NA | NA | NA | NA |
| client_insits_antibiotic | 0.2820068 | 0.07188299 | 3.92313662 | 0.04932489 | 5.71733276 |
| gastro_side_effects | 0.12637457 | 0.0594345 | 2.1262831 | 0.04279626 | 2.95293475 |
| gastro_side_effects_5 | 0 | NA | NA | NA | NA |

Table S3: Estimates from DCE: SAP. Std.err - standard error, t-ratio(0) – t-ratio versus 0, Rob.std.err - robust standard error, Rob t-ratio(0) – robust t-ratio versus 0. Number of respondents = 1,093; final log-likelihood = -6,146.60; null log-likelihood = -7,204.70; AIC = 12,341.21; BIC = 12,504.13; McFadden's pseudo-R-squared = 0.1469.

|  | Estimate | Std.err. | t-ratio(0) | Rob.std.err. | Rob.t-ratio(0) |
| --- | --- | --- | --- | --- | --- |
| scale_english | 1 | NA | NA | NA | NA |
| scale_spanish | 0.94934576 | 0.13513746 | 7.02503782 | 0.21825188 | 4.34977126 |
| scale_portuguese | 1 | NA | NA | NA | NA |
| scale_chinese | 0.722674 | 0.07227424 | 9.999053 | 0.12035825 | 6.0043579 |
| scale_japanese | 1 | NA | NA | NA | NA |
| scale_turkish | 1 | NA | NA | NA | NA |
| scale_vietnamese | 1 | NA | NA | NA | NA |
| scale_romanian | 1 | NA | NA | NA | NA |
| three_five_day_antibiotic | 0.78297927 | 0.10692561 | 7.32265406 | 0.18232962 | 4.29430654 |
| seven_day_antibiotic | 0 | NA | NA | NA | NA |
| length_male | -0.0920223 | 0.06787503 | -1.355761 | 0.14812979 | -0.6212276 |
| length_years_practice | -0.0003504 | 0.00307857 | -0.11381 | 0.00662443 | -0.0528909 |
| length_diff_country_practice_to_trained | 0.12152133 | 0.09682671 | 1.2550394 | 0.2095446 | 0.57993062 |
| length_Africa | 0.56985186 | 0.30898287 | 1.844283 | 0.70087644 | 0.81305609 |
| length_Asia | 1.25717603 | 0.12051094 | 10.4320494 | 0.23116168 | 5.43851409 |
| length_Australasia | 0.35225353 | 0.15572007 | 2.26209461 | 0.32142749 | 1.09590356 |
| length_NorthernEurope | -0.5809089 | 0.13074903 | -4.4429311 | 0.25968553 | -2.2369707 |
| length_EasternEurope | 0.60781538 | 0.12113128 | 5.0178236 | 0.24769969 | 2.45383992 |
| length_SouthernEurope | 0.90588632 | 0.10202317 | 8.87922128 | 0.21550363 | 4.20357797 |
| length_WesternEurope | -0.8847164 | 0.19674096 | -4.4968594 | 0.38615941 | -2.2910653 |
| length_NorthAmerica | 0.51548155 | 0.1431569 | 3.60081529 | 0.30485415 | 1.69091202 |
| length_SouthAmerica | 1.57866579 | 0.20019256 | 7.88573658 | 0.35623144 | 4.43157347 |
| length_Other | 0 | NA | NA | NA | NA |
| clinical_cure_rate_minus_20 | -1.0676994 | 0.1573312 | -6.7863172 | 0.1591007 | -6.7108406 |
| clinical_cure_rate_minus_15 | -0.1687851 | 0.05358404 | -3.149914 | 0.03796473 | -4.4458406 |
| clinical_cure_rate_minus_10 | 0.0033659 | 0.06051879 | 0.0556175 | 0.03761508 | 0.08948282 |
| clinical_cure_rate_minus_5 | 0 | NA | NA | NA | NA |
| microbiological_cure_rate_minus_20 | -0.4678971 | 0.07040392 | -6.6458946 | 0.08307125 | -5.6324789 |
| microbiological_cure_rate_minus_15 | -0.1106893 | 0.05974635 | -1.8526545 | 0.05025398 | -2.2025984 |
| microbiological_cure_rate_minus_10 | 0.00511982 | 0.05731296 | 0.08933098 | 0.05108731 | 0.10021711 |
| microbiological_cure_rate_minus_5 | 0 | NA | NA | NA | NA |
| gastro_side_effects_1 | 0.12774886 | 0.04687711 | 2.72518648 | 0.04979229 | 2.56563511 |
| gastro_side_effects_5 | 0 | NA | NA | NA | NA |
| client_follows_recommendation | 0 | NA | NA | NA | NA |
| client_insits | -0.1229795 | 0.08787877 | -1.3994219 | 0.09872865 | -1.245631 |
| clinical_signs_none | 0 | NA | NA | NA | NA |
| clinical_signs_moderate | -0.290665 | 0.0921527 | -3.1541666 | 0.10400372 | -2.7947555 |
| clinical_signs_severe | -0.7651732 | 0.08822192 | -8.6732773 | 0.08252228 | -9.2723216 |

Table S4: Estimates from DCE: UTI. Std.err - standard error, t-ratio(0) – t-ratio versus 0, Rob.std.err - robust standard error, Rob t-ratio(0) – robust t-ratio versus 0. Number of respondents = 1,042; final log-likelihood = -6,332.88; null log-likelihood = -6,866.33; AIC = 12,715.76; BIC = 12,884.27; McFadden's pseudo-R-squared = 0.0777.
